# cAMP-stimulated Cl^-^ secretion is increased by glucocorticoids and inhibited by bumetanide in semicircular canal duct epithelium

**DOI:** 10.1186/1472-6793-13-6

**Published:** 2013-03-27

**Authors:** Satyanarayana R Pondugula, Suresh B Kampalli, Tao Wu, Robert C De Lisle, Nithya N Raveendran, Donald G Harbidge, Daniel C Marcus

**Affiliations:** 1 Dept. Anatomy & Physiology, Cellular Biophysics Laboratory, Kansas State University, Manhattan, KS 66506, USA; 2 Dept. Anatomy & Cell Biology, University of Kansas Medical Center, Kansas City, KS 66160, USA

**Keywords:** Chloride secretion, Rat, Knockout mouse, Primary culture, Epithelium, Inner ear, Bumetanide, DIOA, Glucocorticoid, NKCC, KCC

## Abstract

**Background:**

The vestibular system controls the ion composition of its luminal fluid through several epithelial cell transport mechanisms under hormonal regulation. The semicircular canal duct (SCCD) epithelium has been shown to secrete Cl^-^ under β_2_-adrenergic stimulation. In the current study, we sought to determine the ion transporters involved in Cl^-^ secretion and whether secretion is regulated by PKA and glucocorticoids.

**Results:**

Short circuit current (*I*_*sc*_) from rat SCCD epithelia demonstrated stimulation by forskolin (EC_50_: 0.8 μM), 8-Br-cAMP (EC_50_: 180 μM), 8-pCPT-cAMP (100 μM), IBMX (250 μM), and RO-20-1724 (100 μM). The PKA activator N6-BNZ-cAMP (0.1, 0.3 & 1 mM) also stimulated *I*_*sc*_. Partial inhibition of stimulated *I*_*sc*_ individually by bumetanide (10 & 50 μM), and [(dihydroindenyl)oxy]alkanoic acid (DIOA, 100 μM) were additive and complete. Stimulated *I*_*sc*_ was also partially inhibited by CFTR_inh_-172 (5 & 30 μM), flufenamic acid (5 μM) and diphenylamine-2,2^′^-dicarboxylic acid (DPC; 1 mM). Native canals of CFTR^+/−^ mice showed a stimulation of I_sc_ from isoproterenol and forskolin+IBMX but not in the presence of both bumetanide and DIOA, while canals from CFTR^−/−^ mice had no responses. Nonetheless, CFTR^−/−^ mice showed no difference from CFTR^+/−^ mice in their ability to balance (rota-rod). Stimulated *I*_*sc*_ was greater after chronic incubation (24 hr) with the glucocorticoids dexamethasone (0.1 & 0.3 μM), prednisolone (0.3, 1 & 3 μM), hydrocortisone (0.01, 0.1 & 1 μM), and corticosterone (0.1 & 1 μM) and mineralocorticoid aldosterone (1 μM). Steroid action was blocked by mifepristone but not by spironolactone, indicating all the steroids activated the glucocorticoid, but not mineralocorticoid, receptor. Expression of transcripts for CFTR; for KCC1, KCC3a, KCC3b and KCC4, but not KCC2; for NKCC1 but not NKCC2 and for WNK1 but only very low WNK4 was determined.

**Conclusions:**

These results are consistent with a model of Cl^-^ secretion whereby Cl^-^ is taken up across the basolateral membrane by a Na^+^-K^+^-2Cl^-^ cotransporter (NKCC) and potentially another transporter, is secreted across the apical membrane via a Cl^-^ channel, likely CFTR, and demonstrate the regulation of Cl^-^ secretion by protein kinase A and glucocorticoids.

## Background

The inner ear controls the ion composition of its luminal fluid, endolymph, through a multiplicity of transepithelial transport mechanisms in different cell types bounding the lumen. The high-K^+^, low Na^+^, low Ca^2+^ endolymph composition is needed for proper auditory and vestibular function [1-3]. K^+^ secretion by both strial marginal cells and vestibular dark cells is stimulated by β-adrenergic receptors via cytosolic cAMP as second messenger [4,5]. Secretion of the primary anion, Cl^-^, is known to also be under adrenergic control in semicircular canal duct (SCCD) epithelium [6].

Cl^-^ transport by several epithelia has been shown to be under control of a cAMP signal pathway that is mediated by apical CFTR Cl^-^ channels via protein kinase A (PKA) [7]. Vectorial transport in those epithelia depends also on basolateral Na^+^-K^+^-ATPase and K^+^ channels as well as basolateral Cl transporters such as the Na^+^-K^+^-2Cl^-^ cotransporter (NKCC1/Slc12a2) [6,8-10]. Cellular cAMP levels and cAMP-mediated processes, including ion transport, depend on cAMP metabolism regulated by the enzymatic activity of anabolic adenylyl cyclase and catabolic phosphodiesterase [11-13].

Glucocorticoids can modify cellular responses via genomic and non-genomic pathways, including regulation of ion transport processes [14-17]. Na^+^ absorption by SCCD has already been demonstrated to be stimulated by glucocorticoids [17,18]. There is considerable evidence in various tissues, including epithelia, that glucocorticoids have long-term (genomic) effects on cAMP metabolism and potentiate cAMP-mediated responses, including ion transport by activation of the glucocorticoid receptor [19-21].

We therefore sought to determine in SCCD a) whether there is a basal constitutive adenylyl cyclase activity driving cAMP-mediated Cl^-^ secretion, b) whether Cl^-^ secretion is enhanced by glucocorticoid hormones via activation of glucocorticoid receptors, c) whether Cl^-^ secretion is mediated through PKA action and d) whether Cl^-^ secretion is mediated by a basolateral NKCC and/or KCC and an apical CFTR Cl^-^ channel. Our findings establish that the SCCD is a site in the inner ear for PKA-mediated Cl^-^ transport, that this transport depends on NKCC1 and another Cl^-^ uptake process (perhaps KCC), that apical CFTR is likely involved and that it is enhanced by glucocorticoid-receptor activation.

## Results

### Adenylyl cyclase activator, cAMP analogs, and phosphodiesterase inhibitors stimulate *I*_*sc*_

**Figure 1:**
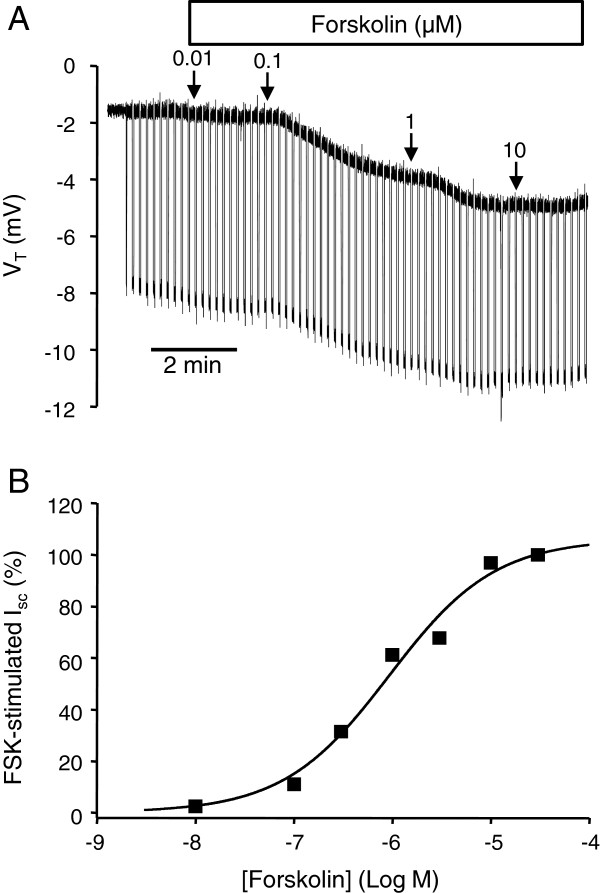
**Stimulation of equivalent short circuit current** (***I***_***sc***_) **by the adenylyl cyclase activator forskolin.** (**A**) Representative recording of V_T_ from primary cultures of SCCD epithelium with the effect of forskolin (0.01 to 10 μM) added cumulatively on both sides at arrows. Downward deflections result from transepithelial current pulses and are proportional to transepithelial resistance. **B**) Summary of concentration-response of *I*_*sc*_ to forskolin (FSK) stimulation (n = 3–5), EC_50_ = 0.8 μM and Hill coefficient 0.9. Summary data are mean ± SEM; error bars are smaller than symbols.

**Figure 2:**
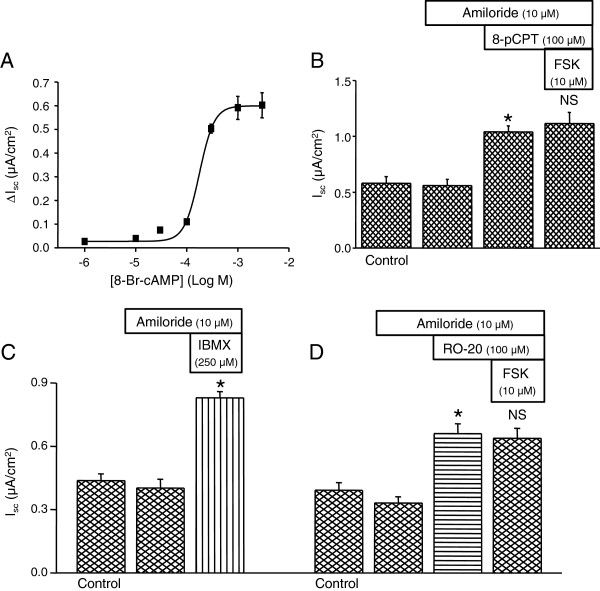
**Membrane-permeable cAMP analogs and phosphodiesterase inhibitors increase *I***_***sc***_**. A**) Summary of concentration-response of *I*_*sc*_ to 8-Br-cAMP (n = 3–4) on both sides after prior application of 10 μM apical amiloride, EC_50_ = 180 μM and Hill coefficient 3.0. **B**) Summary of response of *I*_*sc*_ to 8-pCPT-cAMP (8-pCPT; 100 μM; n = 4) on both sides in the presence of apical amiloride (10 μM); no further stimulation by subsequent forskolin (FSK, 10 μM). **C**) Summary of response of *I*_*sc*_ to 3-isobutyl-1-methylxanthine (IBMX; 250 μM; n = 3) on both sides and **D**) RO-20-1720 (RO-20; 100 μM; n = 3) on both sides after prior application of apical amiloride (10 μM). Summary data are mean ± SEM; *, P < 0.05; NS, not significant; compared to bar immediately to the left.

The SCCD epithelium actively contributes to endolymph homeostasis by Cl^-^ secretion under control of β_2_-adrenergic receptors via a cAMP pathway [6], like other epithelia that are known to secrete Cl^-^ upon stimulation by β-adrenergic receptor activation [22-26]. Forskolin (adenylyl cyclase activator) [6] (Figure 1A & B), cell permeable cAMP analogs (8-Br-cAMP and 8-pCPT-cAMP [100 μM]) (Figure 2A & B), the non-selective phosphodiesterase inhibitor 3-isobutyl-1-methylxanthine (IBMX; 250 μM) (Figure 2C), and the cAMP-specific phosphodiesterase-4 (PDE4) inhibitor RO-20-1724 (100 μM) (Figure 2D) increased *I*_*sc*_ in the presence of apical amiloride (10 μM), an inhibitor of the epithelial Na^+^ channel. The maximal forskolin-stimulated *I*_*sc*_ was 0.58 ± 0.06 μA/cm2 (n=38) (Figure 1B). In the present series of experiments (Figures 2B,C,D), amiloride produced no significant changes in *I*_*sc*_ in the absence of steroids, although in a previous larger series of experiments there was a small (15%) but significant decrease in *I*_*sc*_[17]. The initial transepithelial resistance (Figure 1B) was 4.7 ± 0.6 kΩ-cm^2^ (n=3) and decreased significantly with 30 μM forskolin to 3.1 ± 0.3 kΩ-cm^2^ (n=3).

The lipid-soluble drugs forskolin, 8-pCPT-cAMP, RO-20-1724, 3-isobutyl-1-methylxanthine (IBMX), were added to both the apical and basolateral baths. Amiloride was added to only the apical side and bumetanide to the basolateral side. Amiloride had no significant effect on *I*_*sc*_, whereas subsequent addition of both forskolin (Figure 1) and 8-Br-cAMP (Figure 2A) increased *I*_*sc*_ in a concentration dependent manner with an EC_50_ of about 0.8 μM and 180 μM respectively. Forskolin showed no additional effect after prior stimulation by either 8-pCPT-cAMP (100 μM) (Figure 2B) or by RO-20-1724 (100 μM) (Figure 2D), demonstrating constitutive activity of adenylyl cyclase in SCCD epithelium.

### Glucocorticoids increase forskolin-stimulated *I*_*sc*_

**Figure 3:**
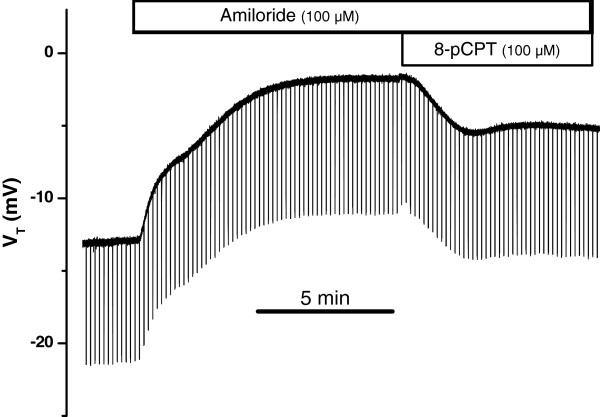
**Stimulation of Cl**^-^**secretion by cAMP after exposure to dexamethasone.** Representative trace of response of V_T_ to apical amiloride and the membrane-permeable cAMP analog 8-pCPT-cAMP on both sides after incubation with dexamethasone (100 nM, 24 h).

We investigated whether forskolin-stimulated Cl^-^ secretion was altered by glucocorticoid treatment (24 hr). As in the absence of dexamethasone, increasing intracellular cAMP in dexamethasone-treated epithelia by exposure to 8-pCPT-cAMP in the presence of amiloride (10 μM) led to an increased *I*_*sc*_ (representative recording in Figure 3). Similar responses were seen with forskolin (10 μM), 8-Br-cAMP (100 μM) and IBMX (250 μM) (data not shown). The glucocorticoid-stimulated Na^+^ absorption via apical sodium channels (ENaC) was blocked by amiloride, which decreased *I*_*sc*_ by 81 – 92% [17]; the remaining current was due to Cl^-^ secretion [6].

**Figure 4:**
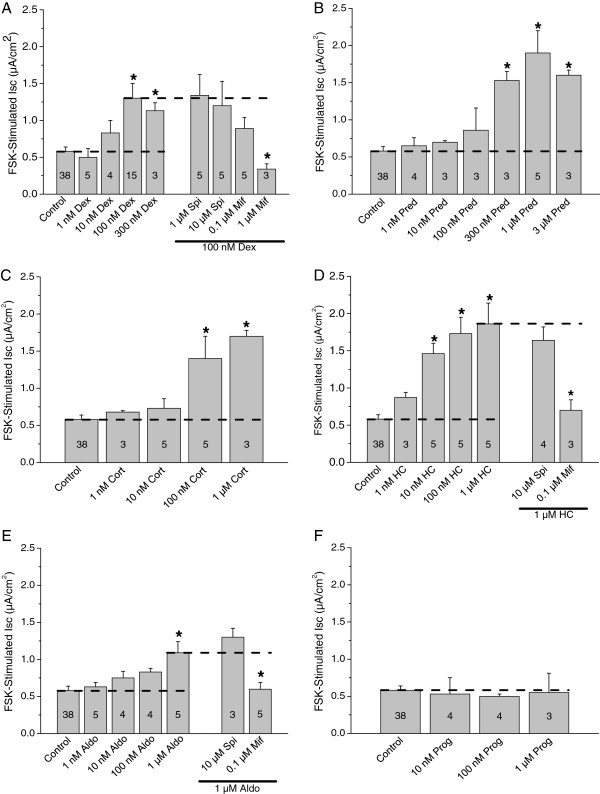
**Response of forskolin-stimulated *I***_***sc***_**to corticosteroids.** Summary data of responses to forskolin (10 μM) after incubation (24 h) in steroids or steroids + receptor antagonists at the concentrations shown. **A**) dexamethasone (Dex) ± spironolactone (Spi) or mifepristone (mif); **B**) prednisolone (Pred); **C**) corticosterone (Cort); **D**) hydrocortisone (HC) ± spironolactone (Spi) or mifepristone (mif); **E**) aldosterone (Aldo) ± spironolactone (Spi) or mifepristone (mif); **F**) progesterone (Prog). The horizontal dashed lines show the reference levels for statistical comparisons.

The concentration-dependence of natural and synthetic glucocorticoids was determined (Figure 4). Interestingly, the stimulation by forskolin was significantly greater after treatment with 100 or 300 nM dexamethasone, as observed previously with single concentrations of dexamethasone and forskolin [17]. Similarly, the stimulation of *I*_*sc*_ by forskolin was significantly greater after 24 hr treatment with the other glucocorticoids (hydrocortisone, corticosterone, and prednisolone) and the mineralocorticoid aldosterone in the continued presence of amiloride (Figure 4). The transepithelial resistance was significantly reduced by about one third after exposure to effective concentrations of glucocorticoids (ANOVA analysis of Table two in [17]), as would be expected after insertion of a conductive pathway (epithelial sodium channels) in the apical membrane.

The natural and synthetic glucocorticoids stimulated within their respective physiologic and therapeutic ranges [17], while aldosterone was only effective at concentrations much higher than found under normal physiologic conditions [17].

### Glucocorticoids increase forskolin-stimulated *I*_*sc*_ by activation of glucocorticoid receptor

We investigated whether dexamethasone, hydrocortisone, and aldosterone increased FSK-stimulated *I*_*sc*_ by activation of glucocorticoid receptors and/or mineralocorticoid receptors. SCCD epithelia were incubated in the presence of dexamethasone (100 nM), hydrocortisone (1 μM) or aldosterone (1 μM) alone or in the presence of receptor antagonists. Mifepristone significantly reduced the effects of dexamethasone, hydrocortisone and aldosterone (Figure 4A,D,E), consistent with action of all of these corticosteroids at the glucocorticoid receptor. Mifepristone is also known to be an antagonist of the progesterone receptor; however, progesterone (10–1000 nM) had no effect on forskolin stimulation (Figure 4F). The mineralocorticoid receptor antagonist spironolactone had no significant effect on dexamethasone-, hydrocortisone- or aldosterone-treated epithelia (Figure 4A,D,E), consistent with a lack of involvement of mineralocorticoid receptor in the increase of the FSK-stimulated *I*_*sc*_ in these cells. These findings suggest that corticosteroids increase forskolin-stimulated *I*_*sc*_ (cAMP-mediated Cl^-^ secretion) solely by activation of glucocorticoid receptors in SCCD epithelia.

### PKA activator stimulates *I*_*sc*_

**Figure 5:**
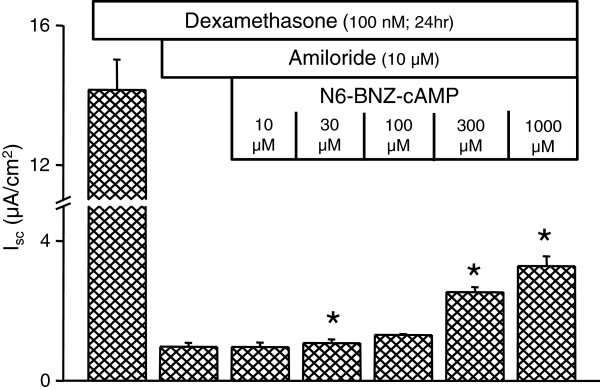
**PKA activation of *I***_***sc***_**.** Summary of response of *I*_*sc*_ to the PKA activator N6-BNZ-cAMP (n = 3) after treatment with dexamethasone in the presence of apical amiloride (10 μM). Summary data are mean ± SEM; *, P < 0.05; compared to bar prior to addition of activator. Note a break in the Y-axis.

It is known that cAMP-dependent Cl^-^ secretion in many epithelia is mostly mediated through activation of protein kinase A (PKA) [7]. We therefore investigated whether PKA activation increases *I*_*sc*_ in SCCD. Indeed, apical and basolateral addition of PKA activator N6-BNZ-cAMP stimulated *I*_*sc*_ at 30, 100, 300 and 1000 μM after prior inhibition of Na^+^ transport with apical amiloride (10 μM) (Figure 5).

### Blockers of basolateral Na^+^-K^+^-2Cl^-^ and K^+^-Cl^-^ cotransporters inhibit forskolin and/or forskolin + IBMX-stimulated *I*_*sc*_

**Figure 6:**
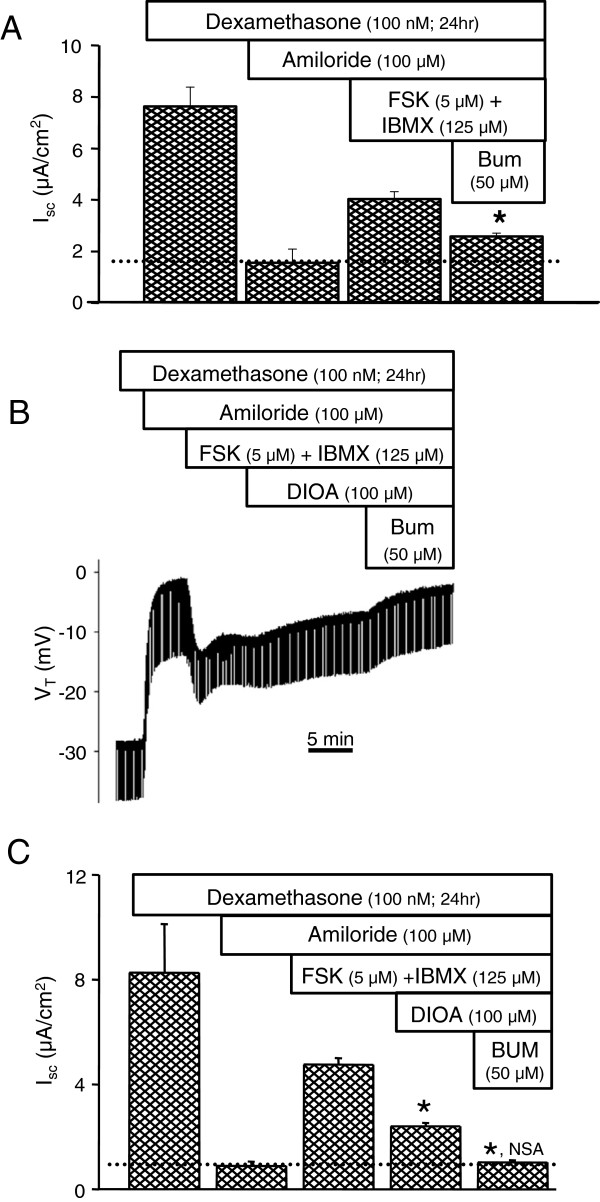
**Inhibition of forskolin-stimulated *I***_***sc***_**by bumetanide and DIOA. A**) Summary of inhibition by basolateral bumetanide (BUM) (n = 11) of cAMP-stimulated *I*_*sc*_ (forskolin, FSK + IBMX). Epithelia were incubated in dexamethasone and Na^+^ transport was inhibited with apical amiloride before stimulation by cAMP. Note that *I*_*sc*_ does not return after only bumetanide to the basal Cl^-^ secretion rate in amiloride (dotted line). **B**) Representative recording of V_T_ and the response to apical amiloride forskolin + IBMX, basolateral DIOA and basolateral bumetanide (BUM). **C**) Summary of response of *I*_*sc*_ to DIOA and bumetanide (BUM) (n = 3) after stimulation by forskolin+ IBMX. Na^+^ transport was inhibited with apical amiloride before stimulation. Note that *I*_*sc*_ returns after both DIOA and bumetanide to the basal Cl^-^ secretion rate in amiloride (dotted line). Summary data (A and C) are mean ± SEM; *, P < 0.05 compared to bar immediately to the left; NSA, not significantly different from amiloride.

We have previously shown that both Ba^2+^-sensitive K^+^ channels and ouabain-sensitive Na^+^-K^+^-ATPase are involved in Cl^-^ secretion by SCCD [6]. However, the participation of Na^+^-K^+^-2Cl^-^ and K^+^-Cl^-^ cotransporters is not known. Therefore, we investigated whether these transport proteins are involved in cAMP-mediated Cl^-^ secretion. Untreated and dexamethasone (100 nM; 24 hr)-treated SCCD epithelia were stimulated with apical and basolateral forskolin and forskolin + IBMX respectively, followed by application of blockers of ion transporters to the basolateral side. Na^+^-K^+^-2Cl^-^ cotransporter inhibitor bumetanide (50 μM) partially decreased the magnitude of forskolin (5 μM) + IBMX (125 μM)-stimulated *I*_*sc*_ (Figure 6A). Bumetanide (10 & 50 μM) also decreased the forskolin (10 μM)-stimulated *I*_*sc*_ by 20 ± 2% (10 μM; n = 8) and 18 ± 5% (50 μM; n = 3) (data not shown). Similarly, the K^+^-Cl^-^ cotransporter blocker [(dihydroindenyl)oxy]alkanoic acid (DIOA, 100 μM) partially inhibited the forskolin + IBMX-stimulated *I*_*sc*_ (Figure 6B). Interestingly, DIOA and bumetanide, when added to the bath cumulatively, completely inhibited all of the forskolin + IBMX-stimulation, returning *I*_*sc*_ to the level observed with amiloride (Figure 6C). These findings are consistent with the presence of Na^+^-K^+^-2Cl^-^ and K^+^-Cl^-^ cotransporters at the basolateral membrane and their participation in Cl^-^ secretion by SCCD epithelium, although DIOA is lipophilic and could cross to a KCC on the apical membrane. However, the observed effect is not likely a result of action of DIOA on an apical KCC since transport of Cl^-^ by an apical KCC would also result in a similar rate of K^+^ secretion, and no transepithelial K^+^ (Rb^+^) flux was detected from cAMP-stimulated SCCD [6].

### Expression of KCC and WNK isoforms

Additive inhibition of *I*_*sc*_ by bumetanide and DIOA suggested dependence of electrogenic Cl^-^ transport in SCCD on expression of NKCC and KCC. The basolateral isoform of NKCC, (NKCC1/Slc12a2 Affymetrix probe set 1367853_at), was shown to be present in a gene array of SCCD and the apical isoform (NKCC2/Slc12a1 Affymetrix probe set 1368548_at) was absent [27]. Not all isoforms of KCC were identified on the gene chips; we tested for expression of transcripts with RT-PCR and observed expression of KCC1, KCC3a, KCC3b and KCC4; but, KCC2 was absent (Table 1).

**Table 1:** RT-PCR demonstration of the presence of KCC1, KCC3a, KCC3b, KCC4 and absence of KCC2 in rat SCCD primary cultures

**Ct, average**						
Tissue	18S	KCC1	KCC2	KCC3a	KCC3b	KCC4
rSCCD		26.4	BT	25.0	27.3	26.7
rat kidney	14.2	25.9	--	23.3	24.5	26.3
rat brain	--	--	22.6	--	--	--
blank	35.0	30.2	BT	BT	32.3	35.8

BT, Below Threshold fluorescence at end of 38 thermocycles. Ct values are based on duplicate runs of 4 samples SCCD and duplicates of control tissue total RNA. The difference between Ct values of duplicates varied by 0.3 ± 0.04 (n=26) with a range from 0.0 to 0.8.

It is known that genetic mutations in the kinase WNK1 and WNK4 cause a disease featuring hypertension and hyperkalemia and the etiology appears to be related to regulation of NKCC and KCC (reviewed in [49]). We observed that SCCD expresses WNK1 but only very low or absent WNK4 (Table 2).

**Table 2:** RT-PCR demonstration of the presence of WNK1 and absence of WNK4 in rat SCCD primary cultures

**Tissue**	**18S**	**WNK1**	**WNK4**
rSCCD	13.4	24.7	31.6
rat kidney	14.7	25.0	26.0
blank	34.4	BT	35.2

Ct, average. BT, Below Threshold fluorescence at end of 38 thermocycles. Ct values are based on duplicate runs of 4 samples SCCD and duplicates of rat kidney as a control tissue. The difference between Ct values of duplicates varied by 0.6 ± 0.09 (n=36) with a range from 0.0 to 2.1.

#### Blockers of apical Cl^-^ transport inhibit forskolin and/or forskolin + IBMX-stimulated I_sc_

**Figure 7:**
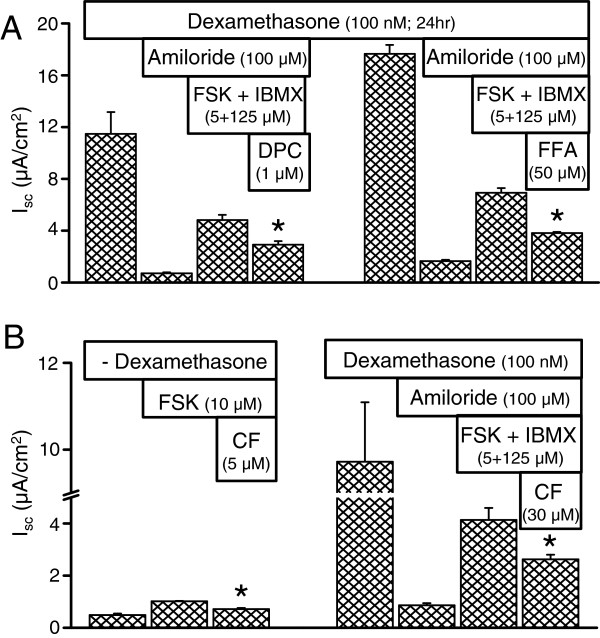
**Partial inhibition of *I***_***sc***_**in rat primary cultures by blockers of CFTR. A**) Diphenylamine-2,2^′^-dicarboxylic acid (DPC) and flufenamic acid (FFA) significantly reduced cAMP-stimulated *I*_*sc*_. **B**) CFTR_inh_-172 significantly reduced cAMP-stimulated *I*_*sc*_ in both the absence and presence of dexamethasone. FSK, forskolin; CF, CFTR_inh_-172. *, P < 0.05.

Cl^-^ secretion across the apical membrane in many epithelia is mediated by the CFTR Cl^-^ channel [8,10]. Na^+^ currents were blocked by amiloride (100 μM) in dexamethasone (100 nM, 24 hr)-treated SCCD epithelia. *I*_*sc*_ was then stimulated with apical and basolateral forskolin (5 μM) + IBMX (125 μM), followed by apical addition of blockers of Cl^-^ transporters. Non-selective Cl^-^ channel inhibitors DPC (1 mM) and flufenamic acid (5 μM) (Figure 7A) partially inhibited the stimulated *I*_*sc*_. The CFTR Cl^-^ channel blocker CFTR_inh_-172 (5 μM or 30 μM) [28] partially inhibited the forskolin (10 μM) and forskolin (5 μM) + IBMX (125 μM)-stimulated *I*_*sc*_ in both the absence and presence of dexamethasone (100 nM, 24 hr; Figure 7B, *left* and *right* panels). Taken together, these results suggest that CFTR Cl^-^ channels are functionally expressed at the apical membrane and account for at least part of the Cl^-^ secretion by SCCD epithelium, although CFTR appears to not be essential for vestibular function under normative conditions (see below). The observation of the presence of mRNA transcripts of CFTR in gene arrays of rat primary cultures (Affymetrix Probe set 1384960_at [27]) is consistent with that interpretation.

**Figure 8:**
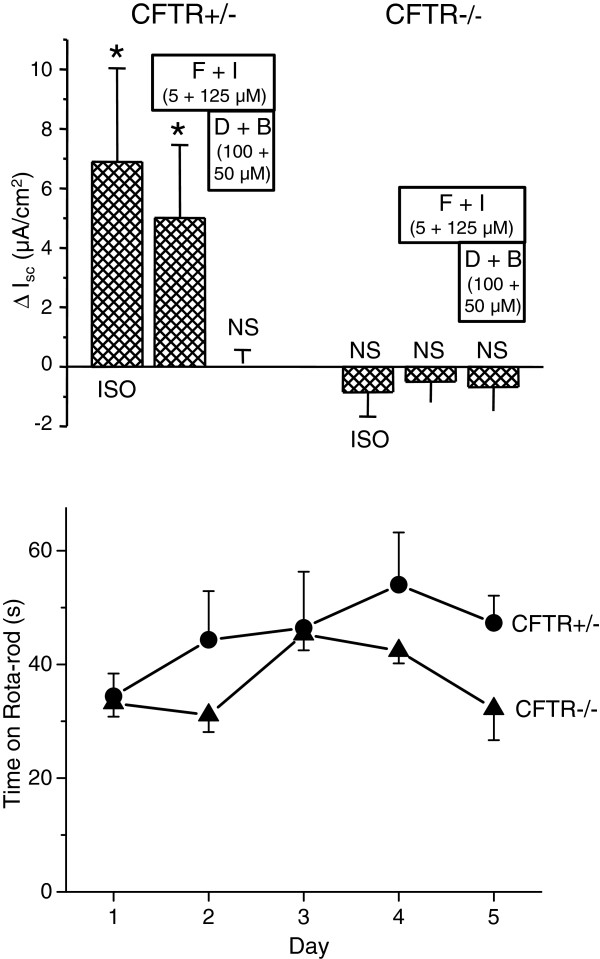
**Short circuit current and balance in CFTR knockout mice.** (*top panel*) Vibrating Probe recordings of *I*_*sc*_ from native SCCD from heterozygote (CFTR+/−) and CFTR knockout (CFTR−/−) mice (n=3 each). ISO, Isoproterennol (10 μM); F, Forskolin; I, IBMX; D, DIOA; B, Bumetanide. The control bath solution contained Amiloride (10 μm). Perfusion was returned to control solution after isoproterenol. *, significant change in current by treatment; P < 0.05. NS, not significant. (*bottom panel*) Balance function of heterozygote (CFTR+/−) and CFTR knockout (CFTR−/−) mice was tested with a rota-rod apparatus. No significant difference was found between the two groups (n=5 each).

SCCD from CFTR^+/−^ mice showed an increase in response of *I*_*sc*_ to a β_2_-adrenergic receptor agonist isoproterenol (100 nM) and a mixture of forskolin and IBMX (Figure 8, *top panel*). Heterozygous CFTR mice are known to have an ion transport profile similar to wild-type mice [29]. A mixture of DIOA (KCC inhibitor) and bumetanide (NKCC inhibitor) completely inhibited the increased *I*_*sc*_ by forskolin + IBMX (Figure 8, *top panel*). This result is consistent with the results shown above in rat SCCD primary cultures (Figure 6C). On the other hand, SCCD from CFTR^−/−^ mice lacked response to both isoproterenol (10 μM) and a mixture of forskolin and IBMX, suggesting that all of the cAMP-stimulated *I*_*sc*_ is mediated by, or dependent on, CFTR in mouse SCCD (Figure 8, *top panel*). The vestibular functional phenotype, as assessed by Rota-Rod, of the CFTR^−/−^ mice did not differ significantly from the CFTR^+/−^ mice (Figure 8, *bottom panel*), in spite of the profound difference in stimulated *I*_*sc*_ (above).

## Discussion

Sensory transduction of acceleration in the vestibular labyrinth is mediated by modulation of K^+^ currents through sensory cells, where the K^+^ originates from the high-[K^+^] luminal fluid. Secretion of K^+^ is controlled by β-adrenergic stimulation (among other signal pathways) [30,31] and it is to be expected that secretion of the primary anion would also be regulated by the same agonists. Movements of anions would be expected to be transcellular since the paracellular pathway must be extremely tight in this epithelium to support the large ion concentration gradients. Cl^-^ secretion by the semicircular canal duct (SCCD) in the vestibular labyrinth is stimulated by cAMP as second messenger [6] and similar mechanisms have been proposed in the cochlea [32,33], although the cell types responsible in the cochlea have not yet been unambiguously determined (see [34]).

The SCCD is an epithelial domain that has a high ratio of surface area to endolymph volume and would therefore be a strong candidate for a site of effective ion homeostasis. Indeed, it was recently shown that these cells also absorb Na^+^ via the epithelial sodium channel (ENaC) under glucocorticoid receptor control [17,18] and absorb Ca^2+^ via an epithelial Ca^2+^ channel [35], in addition to their role in Cl^-^ secretion.

We determined in the present study that cAMP acts via PKA, whose target may be an apical CFTR Cl channel [6]. Evidence supporting the involvement of CFTR include inhibition of Cl^-^ secretion (cAMP-stimulated *I*_*sc*_) by the poorly-specific inhibitors flufenamic acid and DPC and by the specific inhibitor CFTR_inh_-172. Partial inhibition in rSCCD by CFTR_inh_-172 is consistent with reports of significant but only partial inhibition in avian proximal tubule at 20 μM [36]. In addition, mRNA message for CFTR was found to be present in the rat primary cultures of SCCD (GEO database, Accession GSE6196, [27]). The observation of cAMP-stimulated *I*_*sc*_ in mouse canals extends the findings in gerbil and rat [6] to another rodent species. The absence of cAMP-stimulated *I*_*sc*_ in CFTR knockout mice is consistent with an essential role of CFTR in canal Cl^-^ secretion, although there is no strict proof ruling out the unlikely occurrence of dissection damage to only the knockout mouse canals.

Interestingly, there is no correlation of deafness in persons with dysfunctional CFTR (cystic fibrosis) [37,38] and no reports of vertigo in this population. Our results with vestibular tests of CFTR knockout mice are consistent with that observation. It may be, however, that this anion transport system is not by itself essential for normal inner ear function, but may be necessary in times of systemic stress when β-adrenergic agonist levels are elevated, but this proposition remains to be tested.

The results here demonstrated increased *I*_*sc*_ by stimulation of the cAMP signal pathway in several ways: through increased cAMP production (forskolin stimulation of adenylyl cyclase), addition of exogenous cAMP analogs (8-Br-cAMP and 8-pCPT-cAMP), and inhibition of cAMP catabolism (IBMX and RO-20-1724 inhibition of phosphodiesterase). The action of all of these agents has been well-documented in other cells [11-13,19-21]. Increased Cl^-^ secretion by inhibition of phosphodiesterase implies that the adenylyl cyclase is constitutively active in the SCCD epithelium in the absence of β-adrenergic agonists, and is therefore consistent with the earlier observation of constitutive low-level Cl^-^ secretion [6]. Stimulation of Cl^-^ secretion has been reported in mouse jejunum, guinea pig distal colon, T84 monolayers and human colonic epithelial cells by phosphodiesterase inhibition [39-42]. Of particular interest is the finding that phosphodiesterase inhibition did not elevate Cl- secretion in the absence of exogenous stimulation of adenylyl cyclase in T84 epithelial monolayers [41]. This is in contradistinction to our observations in rat semicircular canal primary cultures (vide infra and [6]).

The responsiveness of adenylyl cyclase and phosphodiesterase to multiple physiological challenges is related to the presence of multiple families of isoforms with tissue-specific localization [11-13]. Ten isoforms of adenylyl cyclase (AC; membranous AC1-9 and soluble AC) have been identified in mammalian cells [11]. In SCCD, the non-selective adenylyl cyclase activator forskolin, which is known to activate all identified adenylyl cyclase isoforms with varying sensitivity [11,43,44], stimulated Cl^-^ secretion. Gene array results showed expression of transcripts for AC2 and AC4 in SCCD among the genes that were tested (AC2, AC3, AC4, AC5, AC6, and AC8) using Affymetrix Rat Genome 230 2.0 Array chips [27]. However, it is not known whether other isoforms that were not represented on the gene array are expressed in SCCD.

Eleven families of phosphodiesterases (PDE1 to PDE11) have been identified in mammalian cells, with each family having several isoforms [13]. Phosphodiesterases show three types of substrate specificity [13]. The PDE4, PDE7, and PDE8 families hydrolyze cAMP specifically. PDE5, PDE6, and PDE9 are cGMP specific and PDE1, PDE2, PDE3, PDE10, and PDE11 are dual substrate phosphodiesterases i.e. they hydrolyze both cAMP and cGMP. In SCCD, both the non-selective phosphodiesterase inhibitor IBMX and the selective PDE4 inhibitor RO-20-1724 [19] stimulated Cl^-^ secretion. Gene array results showed expression of the transcripts for PDE4 and PDE7 isoforms but not PDE8 in SCCD [27]. Expression of PDE4 at the transcript level is consistent with RO-20-1724 stimulation of *I*_*sc*_.

Glucocorticoids are known to affect cAMP metabolism and potentiate cAMP-mediated responses, including salt and water transport by activation of the glucocorticoid receptor [16,19-21]. It is known that glucocorticoids stimulate anion transport, including Cl^-^ in mammalian ileal mucosa, by increasing the concentration of cyclic nucleotides [16]. Glucocorticoids elevate cellular cAMP levels by regulating the activity of both adenylyl cyclases and phosphodiesterases, predominantly by suppressing the activity of phosphodiesterases [19,20]. It is not known if glucocorticoids increase cAMP-mediated Cl^-^ secretion in SCCD epithelium by a similar mechanism.

Glucocorticoids increase cAMP-mediated Cl^-^ secretion in SCCD epithelium by activation of exclusively the glucocorticoid receptor. However, the exact mechanism of potentiation of cAMP response is not known. The stimulation of cAMP-mediated *I*_*sc*_ could not have been a result of the increase in glucocorticoid-activated ENaC function [17], since increased Na^+^ conductance would depolarize the cell, which in turn would reduce the outward driving force for Cl^-^ across the apical membrane, in contrast to observations. In addition, Na^+^ absorption was blocked by amiloride in these experiments. Another putative mechanism of action of glucocorticoids in SCCD is regulation of NKCC expression, as observed in vascular smooth muscle and trabecular meshwork cells [45,46]. However, there was no change in expression of NKCC1 in SCCD after incubation with 100 nM dexamethasone for 24 h [27]. The molecular mechanism for glucocorticoid-increased cAMP-mediated Cl^-^ secretion in SCCD epithelium has not yet been resolved.

One pathway of corticosteroid action is via the kinases SGK1 and WNK1 [47] among other kinases involved in regulation of ion transport such as SPAK and OSR1 [48]. Our gene array of rat SCCD cultures [27] showed SPAK or Stk39 to be present (1387059_at based on Genbank NM_019362) and minimally down-regulated 1.2-fold by dexamethasone. There were no probe sets identified on the chips for OSR1. WNK1 is downstream from SGK1 and is implicated in the control of both NKCC and KCC transporters and thereby their roles in Cl^-^ transport [49]. The expression of WNK1 in the SCCD may therefore be involved in the regulation of Cl^-^ secretion in SCCD through these two transporters. The transcript expression for WNK4 was nearly absent.

Complete additive inhibition of the cAMP-stimulated *I*_*sc*_ by bumetanide and DIOA is consistent with the participation of both Na^+^-K^+^-2Cl^-^ and K^+^-Cl^-^ cotransporter in Cl^-^ cotransport. The concentration of bumetanide used fully inhibits NKCC1 but has no effect on KCC1 and KCC4 [50,51]. Similarly, DIOA is a potent well-established KCC transport inhibitor (IC50 = 10–40 μM) that is without effect on NKCC [52,53] and has been used as a specific KCC inhibitor in the range 30 – 100 μM [53-55]. However, DIOA has also been observed in several epithelia to have diverse effects [56-59].

The canonical interpretation that the effect of DIOA is a result of inhibition of a KCC, however, is problematic. Foremost, the KCC would be operating “backwards” from its most-commonly observed mode and if this were indeed the case it would necessitate an extremely low intracellular [Cl^-^]. A low intracellular concentration of the secreted ion species (Cl^-^) would not favor a strong efflux across the apical membrane. Although most cells operate KCC transporters as cellular *efflux* pathways, Payne calculates that some neuronal cells have sufficiently-low intracellular [Cl^-^] to thermodynamically drive *influx* of KCl [60]. The membrane potential, intracellular [K^+^] and [Cl^-^] of SCCD epithelial cells is not known, so it is not possible at this time to evaluate whether these cells are able to support concurrent basolateral KCC electroneutral influx and apical electrogenic Cl^-^ secretion. The discussion by Payne [60] shows that at the normal extracellular [K^+^] of about 4 mM, the intracellular [Cl^-^] would need to be about 5 mM or less; a highly unlikely constellation. An elevation of extracellular [K^+^] at the canal epithelium due to hair cell stimulation is also highly unlikely since the hair cells are located remotely from the canals and excess K^+^ would be removed from perilymph by vestibular dark cells recycling the K^+^ back into the lumen.

The observation that basolateral bumetanide causes only a partial inhibition of cAMP-stimulated Cl^-^ secretion at 50 μM suggests the existence of a parallel Cl^-^ uptake mechanism. Block of the remaining secretion by DIOA points to a KCC, subject to the caveats given above. An alternative explanation is that DIOA is not a specific inhibitor for KCC in SCCD and either blocks another Cl^-^ uptake mechanism that operates under thermodynamically-favorable conditions or acts broadly on active transport through inhibition of metabolic processes. This last suggestion has been observed with another lipid-soluble weak acid, the Cl^-^ channel blocker NPPB, restricting its use to <100 μM [61]. In addition, DIOA has been shown to interfere with ATP recovery after metabolic insult and to release cytochrome c from isolated mitochondria at the concentration used here [62]. Nonetheless, if this is the basis for action of DIOA in the semicircular canal epithelium, the metabolic interference is not complete (Figure 6) and the likely involvement of a parallel Cl^-^ uptake pathway in rat canal remains.

**Figure 9:**
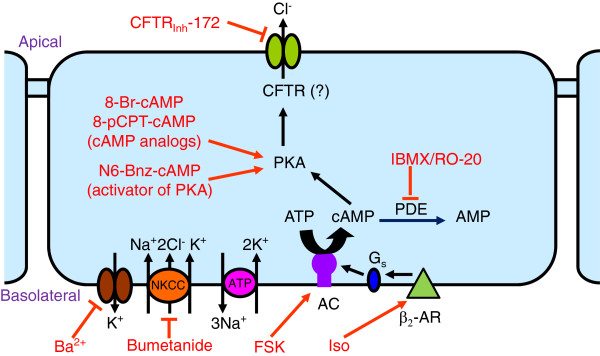
**Proposed cell model for cAMP**/**PKA**-**mediated Cl**- **secretion by SCCD epithelium.** Cl^-^ is taken up across the basolateral membrane by NKCC (Na^+^-K^+^-2Cl^-^ cotransporter). A Na^+^-K^+^-ATPase and Ba^2+^-sensitive K^+^ conductance in the basolateral membrane is thought to create a negative cell membrane potential that drives Cl^-^ exit across the apical membrane via a Cl^-^ channel, likely CFTR. Cl^-^ secretion is stimulated by PKA from activation of a basolateral β_2_-adrenergic receptor (β_2_-AR; activated by agonists such as isoproterenol, ISO) coupled to a cAMP second messenger pathway (G_s_-protein activation of adenylyl cyclase (AC) which can also be directly activated by forskolin (FSK). Phosphodiesterase (PDE) catabolizes cAMP and can be inhibited by IBMX and RO-20-1720 (RO-20). The cAMP-dependent Cl^-^ secretion pathway is further stimulated by activation of intracellular glucocorticoid receptors (not shown) after chronic exposure to glucocorticosteroids.

In summary, our results are consistent with the following cell transport model of regulated Cl^-^ secretion by SCCD (Figure 9). A basolateral Na^+^-K^+^-ATPase removes Na^+^ from the cell and brings in K^+^, which is energized by ATP. The resulting Na^+^ concentration gradient drives the basolateral influx of K^+^ and Cl^-^ along with the recirculating Na^+^ ions via the bumetanide-sensitive NKCC1. Cl^-^ would be secreted across the apical membrane through Cl^-^ channels that may include CFTR as a channel or transport modulator. The rate of secretion is controlled by glucocorticoids and β_2_-adrenergic signalling via cAMP and PKA.

## Conclusions

The rat semicircular canal duct epithelium expresses three major ion transport systems, including a β_2_-adrenergic receptor-stimulated Cl^-^ secretion [6]. We have demonstrated here that Cl^-^ secretion is under control of a PKA-mediated signal pathway that depends on basolateral Cl^-^ uptake via NKCC and (KCC?) transporters and that it is enhanced by activation of glucocorticoid receptors. Interestingly, NKCC does not appear to contribute to ENaC-mediated Na^+^ absorption [17] in the same cells. The role of the β_2_-adrenergic receptor/cAMP pathway in inner ear function remains unknown but is likely activated under stress.

## Methods

All experiments were performed on primary cultures of rat SCCD, except the measurements of short circuit current from native canals and vestibular function that were performed on CFTR mutant mice.

### Cell cultures of rat SCCD epithelium

Primary cultures of neonatal Wistar rat SCCD epithelium were produced as described previously [6]. Briefly, animals were anesthetized and sacrificed according to a protocol approved by the Kansas State University Institutional Animal Care and Use Committee. SCCD epithelial cells were microdissected from isolated temporal bones. Isolated cells, exclusive of connective tissue and common crus, were seeded on 6.5 mm diameter permeable (4 μm) Costar (#3470) Transwell supports and cultured in DMEM/F12 medium (Invitrogen 12500–062; http://www.invitrogen.com/site/us/en/home/support/Product-Technical-Resources/media_formulation.60.html) supplemented with 5% fetal bovine serum, 26.8 mM NaHCO_3_^-^, penicillin (100 U/ml) and streptomycin (100 μg/ml). Cultures treated with steroids, in the presence and absence of antagonists, were exposed for 24 h unless otherwise stated.

### Reagents

Amiloride (#A-7410, Sigma), forskolin (#F-6886, Sigma), spironolactone (#S-3378, Sigma), mifepristone (#M-8046, Sigma), corticosterone (#C-2505, Sigma), prednisolone (#6004, Sigma), bumetanide (#B-3023, Sigma), RO-20-1724 (#557502, Calbiochem), 3-isobutyl-1-methylxanthine (IBMX; #I-7018, Sigma), flufenamic acid (#F-9005, Sigma), CFTR _inh_-172 ([28](gift from Dr. Bruce Schultz), and [(dihydroindenyl)oxy]alkanoic acid (DIOA; # D-129, Sigma) were dissolved in DMSO. Progesterone (#P-8783, Sigma), and aldosterone (#215360050, Acros Organics, New Jersey) were dissolved in absolute ethanol. DMSO or ethanol alone had no effect on electrical parameters in Ussing chamber experiments.

Cyclodextrin-encapsulated dexamethasone (#D-2915, Sigma), hydrocortisone (#H-0396, Sigma), isoproterenol (#I-6504, Sigma), 8-Br-cAMP (#203800, Calbiochem), 8-pCPT-cAMP (#C-3912, Sigma), and N^6^-Benzoyl-Adenosine 3^′^,5^′^-cyclic Monophosphate, Sodium Salt (N6-BNZ-cAMP; #116802, Calbiochem) were dissolved in water.

#### Mice breeding and maintenance

CFTR heterozygous parents in a C57BL/6 background were bred and the offspring were genotyped for CFTR^+/+^, CFTR^+/−^ or CFTR^−/−^ All the animals were maintained on Colyte in water to avoid intestinal impaction [63].

#### Dissection of mouse SCCD

CFTR^+/−^ and CFTR^−/−^ mice (34–39 days old) [63] were anesthetized with sodium pentobarbital (50–100 mg/kg; i.p.) and sacrificed under a protocol approved by the Institutional Animal Care and Use Committee of Kansas State University. The temporal bones were removed and SCCD without common crus were dissected in HEPES-buffered saline. The SCCD were mounted in a 200 μl perfusion chamber on the stage of an inverted microscope (Nikon ECLIPSE TE 300) and continuously perfused at 37°C with HEPES-buffed solution at an exchange rate of 180 μl/sec.

### Electrophysiological measurements

#### I_sc_ from Primary cultures of rat SCCD

SCCD epithelia were bathed in HEPES-buffered solution equilibrated with air. The composition was (in mM) 150 NaCl, 3.6 KCl, 1 MgCl_2_, 0.7 CaCl_2_, 5 glucose, and 10 HEPES, pH 7.4. Transepithelial voltage (V_T_) and resistance (R_T_) were measured from confluent monolayers of SCCD in an Ussing chamber under current clamp (ΔI=1 μA) mode as described previously [17,18]. The equivalent *I*_*sc*_ was calculated from *I*_*sc*_ = V_T_/R_T_. *I*_*sc*_ was measured either in the absence of steroids (Figure 1, 2, 7B *left*) or after 24 h incubation with steroid. Steroid-stimulated Na^+^ current was blocked with amiloride before measurement of cAMP-stimulated *I*_*sc*_. Cytosolic cAMP levels were raised by stimulation of adenylyl cyclase with forskolin, block of phosphodiesterase with IBMX or RO-20-1724 or a combination of forskolin and IBMX, as indicated. The lipid-soluble drugs dexamethasone, hydrocortisone, aldosterone, forskolin, 8-pCPT-cAMP, N6-BNZ-cAMP, spironolactone, mifepristone, corticosterone, prednisolone, progesterone and [(dihydroindenyl)oxy]alkanoic acid (DIOA) were added to both the apical and basolateral baths. Amiloride was added to only the apical side and bumetanide to the basolateral side.

#### I_sc_ from mouse native canal ducts; Voltage-sensitive vibrating probe recordings

The vibrating probe technique was identical to that previously described [34,64]. Briefly, the current density was monitored from mouse SCCD by vibrating (200–400 Hz) a platinum-iridium wire microelectrode with a platinum-black tip positioned 20 μm from the basolateral surface of the SCCD with computer-controlled stepper-motor manipulators (Applicable Electronics, Forest Dale, MA) and probe software (ASET version 2.00, Science Wares, East Falmouth, MA). The bath references were 26-gauge Pt-black electrodes. The phase-locked signal from the electrode was detected by phase-sensitive amplifiers, digitized y (0.5 Hz, 16 bit), and the output was expressed as current density at the electrode. HEPES-buffered solution was used as the bath.

#### Vestibular function test

4 sets of littermate-matched CFTR^+/−^ and CFTR^−/−^ mice (mean age = 56 d) were tested 4 times/day (with a five minute rest between tests) over five days. The RotaRod (Series 8, IITC, Woodland Hills, CA) used a 32 mm diameter rod and was programmed to start at 4 RPM, increasing to 40 RPM over 1 minute. All mice were placed on the rod before rotation was initiated and the time until falling was recorded. The instrument was modified by placing a landing cushion on the platform of the timer stop switch. The daily mean time for each mouse was used to calculate the mean and standard error of each group. After the last run of each day, all homozygous mice were given a 1 ml saline injection to prevent dehydration.

### RNA isolation and RT-PCR

Total RNA was extracted from SCCD primary cultures using RNeasy Micro Kit and the quality and quantity were determined as described previously [18]. RT-PCR was performed on total RNA as described previously using a One Step RT-PCR Kit following the manufacturer’s protocol (#210210, Qiagen) [18].

Gene-specific primers for KCC and WNK isoforms were based on GenBank sequences (Table 3) and were verified to amplify the intended targets. Reverse transcription (RT) was performed on 10 ng of total RNA for 30 min at 50°C and 15 min at 95°C. RT was followed by 38 PCR cycles. Each PCR cycle consisted of 95°C for 1 min, 55°C for 1 min and 72°C for 1 min. To exclude the possibility of genomic DNA amplification during the PCR reaction, RT negative controls were performed (−RT). PCR products were run on 2% agarose gels and detected by ethidium bromide. Purified PCR products were sequenced to verify the identity of the RT- PCR products.

**Table 3:** Gene-specific primers

**Gene**	**Primers 5**^**′**^**—3**^**′**^	**GenBank # (species)**	**Amplicon size (bp)**	**Source**
18S	S: GAG GTT CGA AGA CGA TCA GA	BK000964 (many)	316	[65]
	AS: TCG CTC CAC CAA CTA AGA AC			
KCC1	S: GTT CGC CTC ACT CTT CCT GGC	U55815 (rat)	419	[66]
	AS: TGG GCC ACC ACA TAC AGG GA			
KCC2	S: CAT CAC AGA TGA ATC TCG GG	U55816 (rat)	213	[66]
	AS: TTC TCT GGG TCT GTC TCC C			
KCC3a	S: CCT CGC CTC CTC ACC TTT GC	AF211854 (mouse)	284	This study
	AS: TCA CTC TGA CGC CAG CCA TTG			
KCC3b	S: AGT AAA AGC CCG GAT TCA GG	AF211855 (mouse)	330	[66]
	AS: ATG AAA GTA CCC ATT TGG GG			
KCC4	S: AGG AAG CTG CTG AGC GCA C	AF087436 (mouse)	443	[66]
	AS: CAG CAT TGT ACA GGT GCA GC			
WNK1	S: ACC AGA AAG CCT CAT GTA AGC C	NM_053794 (rat)	301	This study
	AS: GTC CGC AGG GAA CGT CAT TG			
WNK4	S: CAC CTC CCG CCG CAA CAG	NM_175579 (rat)	347	This study
	AS: TCC ACA CAG CAA AGA GCA CCC			

Primers designed on mouse sequences also specifically recognized and amplified the corresponding rat genes. All primer sets were validated to specifically recognize their targets.

### Statistical analyses

Electrophysiology data are presented as original recordings and as mean values ± SE from n observations. Student’s *t*-test was used to determine statistical significance of paired and unpaired samples. The Hill equation was fitted to concentration-response curves by using individual data points in order to retain appropriate weighting and presented here plotted with the mean and SEM. Differences were considered significant for *P* < 0.05.

## Competing interests

The authors declare that they have no competing interests.

## Authors’ contributions

SRP contributed to the design and analysis of the experiments, to performance of many of the electrophysiological measurements and to writing the manuscript. SBK contributed to the design and analysis of the experiments, and to performance of many of the electrophysiological measurements. TW performed the vibrating probe experiments on the CFTR mutant mice. RCD provided the CFTR mutant mice and consulted on their breeding, care and genotyping. NNR designed PCR primers and performed the RT-PCR measurements. DGH contributed to the electrophysiological measurements. DCM contributed to the design and analysis of the experiments and to writing the manuscript. All authors read and approved the final manuscript.

## Authors’ information

Current address for SRP: 112 Greene Hall, Department of Anatomy, Physiology and Pharmacology, College of Veterinary Medicine, Auburn University, Auburn, AL 36849.
